# Educational Case Studies: Creating a Digital Twin of the Production Line in TIA Portal, Unity, and Game4Automation Framework

**DOI:** 10.3390/s23104977

**Published:** 2023-05-22

**Authors:** Michal Balla, Oto Haffner, Erik Kučera, Ján Cigánek

**Affiliations:** Faculty of Electrical Engineering and Information Technology, Slovak University of Technology, 812 19 Bratislava, Slovakia; michal.balla@stuba.sk (M.B.); erik.kucera@stuba.sk (E.K.);

**Keywords:** digital twin, Industry 4.0, digital factory, game4Automation, Unity engine, digital shadow

## Abstract

In today’s industry, the fourth industrial revolution is underway, characterized by the integration of advanced technologies such as artificial intelligence, the Internet of Things, and big data. One of the key pillars of this revolution is the technology of digital twin, which is rapidly gaining importance in various industries. However, the concept of digital twins is often misunderstood or misused as a buzzword, leading to confusion in its definition and applications. This observation inspired the authors of this paper to create their own demonstration applications that allow the control of both the real and virtual systems through automatic two-way communication and mutual influence in context of digital twins. The paper aims to demonstrate the use of digital twin technology aimed at discrete manufacturing events in two case studies. In order to create the digital twins for these case studies, the authors used technologies as Unity, Game4Automation, Siemens TIA portal, and Fishertechnik models. The first case study involves the creation of a digital twin for a production line model, while the second case study involves the virtual extension of a warehouse stacker using a digital twin. These case studies will form the basis for the creation of pilot courses for Industry 4.0 education and can be further modified for the development of Industry 4.0 educational materials and technical practice. In conclusion, selected technologies are affordable, which makes the presented methodologies and educational studies accessible to a wide range of researchers and solution developers tackling the issue of digital twins, with a focus on discrete manufacturing events.

## 1. Introduction

Over the previous few decades, digital manufacturing has added significant value to the whole industry. Digital manufacturing creates models and simulates the development of products and processes by virtually simulating factories, resources, labour forces, and their skills, etc. [1]. The development of manufacturing has been considerably enhanced by the developments in information and communication technologies (ICTs).

The development of smart products and systems that incorporate embedded systems and Internet of Things (IoT) technology, also known as Cyber-Physical Systems (CPS), requires close collaboration between various engineering disciplines. This involves the use of engineering processes to define the products or modules/systems, as well as the production systems needed to create them [2]. Wireless sensor networks, edge computing, cloud computing, the fifth-generation cellular network (5G), IoT, Big data, and other technologies are continually growing and have enormous potential in every industry field [3,4,5,6,7,8]. All of these technologies enable the integration of the physical and digital worlds, which is an unavoidable trend in order to address the market’s increasing complexities and high demands. However, the full strategic benefit of this integration is not being fully realised. The integration process is still in its early stages, with the latest developments focusing on digital twins.

Hribernik et al. [9] introduced the concept of “product avatar” in 2006, which is similar to the concept of a digital twin. From a product-centric point of view, the product avatar concept aimed to build an information management architecture that supports bidirectional information flow. Just before 2015, research on product avatar can be found in [10,11,12]. However, it appears that the product avatar concept has since been replaced by the digital twin.

The digital twin concept is credited to Michael Grieves and his collaboration with NASA’s John Vickers, with Grieves proposing the concept in a presentation on product life-cycle management in 2003 [13]. Grieves and Vickers imagined a world in which a virtual model of a product would set the foundation for product life-cycle management at a time when virtual product representations were “…relatively new and immature” and data about physical products were “…limited, manually collected, and mostly paper-based”.

The initial definition of a digital twin is a virtual representation of a physical product that contains information about that product, with its roots in the field of product life-cycle management. A physical product, a virtual representation of that product, and the bi-directional data connections that transfer information and processes from the physical to the virtual representation and back again are the three elements that make up the digital twin, according to the paper [13], which elaborates on this definition. This flow was represented as a cycle between the physical and virtual states, with data moving from the physical to the virtual and information and processes moving from the virtual to the physical (also known as mirroring or twinning) [14].

The review [15] described three potential applications for digital twins: managing the entire life cycle of the physical object, managing health conditions to plan maintenance activities, and enhancing decision-making through engineering and numerical analysis.

**Figure 1:**
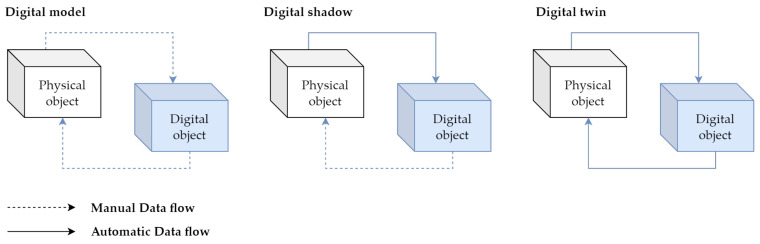
The difference between digital model, digital twin, and digital shadow.

The digital twin concept in manufacturing was examined from a categorical perspective by [16]. The author proposed three subcategories of digital twin based on data-integration levels to clarify the level of digital twin integration in existing studies. To define the depth of integration of the digital twins in existing studies, the author proposed three subcategories of digital twins based on data-integration levels: **digital model**, **digital shadow**, and **digital twin**. We can see all three categories in Figure 1.

A digital model is defined as a digital copy of an existing or planned physical object; there must be no automated data exchange between the physical model and digital model to define a digital model accurately [17]. Plans for constructions, product designs, and development are a few examples of digital models. The important distinctive feature is that there is no automatic data exchange between the physical system and digital model. This means that, after the digital model is created, any changes made to the physical object have no influence on the digital model.

A digital shadow is a one-way flow of information between a physical and digital object. A change in the physical object’s state causes a change in the digital object, not the other way around [18].

The reference “digital twin” is formed when data flows between an existing physical object and a digital object, and they are fully integrated in both directions. A change to the physical object triggers a change in the digital object, and vice versa [19].

These three definitions assist in identifying the typical misunderstandings found in the literature. However, a number of myths are frequent; they are not just seen in these limited scenarios. One of the misconceptions is that digital twins must be an exact 3D model of a physical object. However, some authors believe that a digital twin is just simply a 3D model [20].

Digital twin technology has found applications in a variety of industries that are undergoing digital transformation. The paper [21] identified ten major industrial sectors where digital twin has been used in their study on digital twin applications. These industries are as follows: (i) Aerospace, (ii) Manufacturing, (iii) Healthcare, (iv) Energy, (v) Automotive, (vi) Petroleum, (vii) Public sector, (viii) Mining, (ix) Marine, and (x) Agriculture. They also stated that digital twin has been used in these industries for three main purposes: simulation, monitoring, and control. However, the applications of digital twins are not limited to just these three. Design, validation, customisation, optimisation, prediction, and maintenance are all now performed with digital twins. [22], the industry which will be leading in digital twin deployment by 2021 is manufacturing (34%), followed by energy (18%). Interesting applications of DT are in papers [23,24] They apply DT in the context of construction industry such as On-Site Construction, Building Lifecycle Management and Infrastructure Operations, Blockchain Technology, Sustainability Assessment, or Prefabrication.

## 2. Related Works

In this section we will discuss the works aimed at using digital twin mainly (but not only) in production and manufacturing processes. The research was focused on works using such technologies as digital twin, Unity, PLC, and virtual reality/augmented reality.

### 2.1. Robotic Application with Digital Twin or Digital Shadow

In this paper [25], a digital twin model is proposed to assist in the online/remote programming of a robotic cell. The model consists of a physical model (a FANUC robot) and a digital model (created in the Unity game engine) that replicates the physical robot. Using a digital twin setup along with virtual reality, the authors observe the trajectory replication between digital and physical robots, with a latency of approximately 40 ms and an error range of −0.3 to 0.3 degrees across the robot joint movements. The model is deemed suitable for industrial applications, as it enables the user to program the robot’s trajectory by writing and modifying code within the digital environment. The digital model is created by importing a CAD file into the Unity platform, where additional details and components are added to create a realistic simulation environment. Communication between the digital and physical models is established using the FANUC robot’s API, allowing the user to control the physical robot through the digital model.

The article [26] presents a method of programming robots using virtual reality and digital twins. The virtual environment is a digital twin of a robotic station, built based on CAD models of existing station elements. The virtual reality system is used to record human movements in a virtual environment, which are then reproduced by a real robot. The method is intended to be used in situations where it is necessary for the robot to reproduce the movements of a human performing a complicated process. An example of using the method to programme a robot to clean ceramic casting moulds is presented.

The aim of the research in [27] is to develop a synchronisation model of real and virtual industrial robots using the Unity game engine. This model will be tested in virtual reality and shop floor labs. The primary outcome of the research is to enable universal control algorithms through a VR experience that can be easily modified for a wide range of industrial equipment. The digital twin of an industrial robot, Motoman GP8, was used for the experimental research and was developed using 3D modelling software. The software is designed to be easily extended to support other VR platforms and control digital twins of different industrial robots and equipment. The research also aims to improve workspace awareness of the real robot using its digital twin. Two scripts were developed for this purpose and were tested in VR and on the shop floor.

Study [28] presents the results of an experiment comparing the use of a traditional physical teach pendant and a virtual reality (VR) interface for controlling an industrial robot. In the digital twin can be operated in “coupled” and “virtual” modes. In the coupled mode, all commands are duplicated and sent to the physical robot over the local network, effectively keeping the virtual robot synchronised with its real-world counterpart. In the virtual mode, the network link between the digital twin and the real robot is disconnected. All actions happen inside the simulation only. Apart from these connection modes, the UI provides two control mechanisms for commanding robot motions. The user can either directly tele-operate the robot arm by adjusting individual joint positions, or create a multi-step, joint-space programme to be stored and executed later.

The Cyber-Physical and Intelligent Robotics Laboratory has been recreated digitally in [29], including all key elements for six-axis industrial robots to perform PTP, LIN, and CIRC motions. The human–machine interface has also been integrated, allowing users to create programmes with these motion types. The digital lab can be used for in-house training for small and medium-sized enterprises, without the need for installation, maintenance, or servicing costs. The customisability and virtual format also mean that there is no capacity limit and trainees can participate in exercises in parallel. Exercises were conducted to evaluate the programme’s impact on teaching and showed that using machine units can improve teaching. The digital lab uses the Unreal Engine 4 and Blender to create a simulation of a robot control method.

### 2.2. Applications of Digital Twin Using VR/AR

The paper [30] discusses the use of digital twin technology in modern manufacturing enterprises. The technology allows for the digital virtualisation of different parts of the production process, which can be monitored and controlled using SCADA systems. The paper proposes the use of AVEVA Wonderware software as a tool for implementing the digital twin concept, with the goal of creating a virtual image of a real production system in the initial stage. It also discusses the importance of following the relations and rules throughout the product life cycle, and the need for sufficient depth and fineness of information about the controlled system to ensure the accuracy of the virtual image.

The article [31] discusses the use of a digital twin and virtual reality (VR) to train operators in Industry 4.0 environments. The research used a VR model developed by Game4automation, which simulates a factory setup and allows participants to control and interact with the machines. The study found that VR and the digital twin can be effective in training workers, and that it is especially helpful for older workers who may have difficulty adapting to new industrial paradigms. Feedback from participants also identified areas for improvement in the VR model, such as the user interface and task instructions.

Methodology for developing low-cost, content-rich augmented reality (AR) software using a liquid-soap synthesis process as a case study is presented in [32]. The method comprises four main modules: data creation and collection, integration, cross-platform development, and digital assets. The data creation and collection module involves creating 3D computer graphics and multiphysics computational fluid dynamics (CFD) simulations. The authors demonstrate the use of the Unity game engine to integrate these simulations into AR software and deploy the resulting tool on a mobile platform. The case study is intended to provide guidance for inexperienced developers looking to create digital teaching content. The digital tool developed in the study is available online.

The authors in [33] present a digital twin of a laser-based assembly assistance system for manual mounting tasks. The digital twin was developed using the Unity 3D game engine and implemented in augmented reality using the Microsoft HoloLens. The authors demonstrate that the digital twin can be used to support the planning, simulation, and training of employees at the shop floor. The digital twin is connected to the Mindsphere cloud and can be controlled using hand gestures and voice commands. The implementation is intended to support the use of digital twins in the planning, simulation, and training of employees in production environments.

The research in [34] is focused on digital game-based examination environment for postgraduate engineering students studying Industry 4.0 topics. The environment was developed using the Unity 3D game engine and is intended to provide a more comprehensive evaluation of students’ skills and knowledge. The authors describe the use of digital game-based learning and gamification in education and present a taxonomy for learning objectives. They also explain the development of the game environment and present results from an evaluation of the environment by students. The authors conclude that the game-based examination provides a useful supplement to traditional exams and can be used to evaluate complex problem-solving and application skills.

The paper [35] discusses a novel approach to establishing a closed-loop engineering process chain for the digital factory using a combination of technologies including Game4Automation, VR, and the Internet of Things (IoT). The authors developed a VR demonstrator using the CONTACT Elements for IoT platform and a web-based production process planner and manufacturing execution system (MES) written in Java. The approach involves creating a digital twin of the production process, allowing for real-time analysis of errors or inefficiencies and simulation of changes to the production line. The demonstrator is a virtual production line and does not involve a real production line. The paper describes the architecture and functionality of the demonstrator, as well as potential applications and future developments of the approach.

The text [36] presents a case study where an AR system accesses a digital twin via web services to display real-time information to the user. The architecture of the proposed system includes five layers: devices, user interfaces, web services, queries, and data repositories. The case study involves the visualisation of an offshore oil platform process, which is a cyber-physical system (CPS) process. The AR application uses image recognition to identify equipment and display information on different virtual models, while the digital twin is updated in real-time through web services.

### 2.3. Applications of Digital Twin for Manufacturing Process Using PLC

This article [37] describes the creation of a digital twin for an experimental assembly system. The system includes a belt conveyor and an automatised line for quality production check. The digital twin is a 3D model created in CAD design software and imported to a Tecnomatix platform to simulate all processes. Data from the assembly system is collected by a programmable logic controller system and synchronised by an OPC server to the digital twin model and a cloud platform. The digital twin allows for online optimisation of the assembly process without stopping the production line.

This work [38] presents a testbed for virtual commissioning of a Siemens PLC system in Unity. The testbed simulates a production process and changes the PLC programme based on its behaviour. The authors describe their creation of a data structure and communication module for connecting the simulation with a future optimisation algorithm. They also report the creation of a functional monitoring system for the simulation and a functional data sending system to a server. The simulation monitoring records all collisions with monitored elements. The work also reports the creation of a PLC process programme, a redesigned simulation programme in Unity, and a new REST-API server with a JSON-formatted data structure.

The authors of [39] discuss the implementation of virtual instruction manuals at mass production workplaces in the automotive industry. A pilot study was conducted using a system that utilises its own software solution for creating instructions and connecting them to a Programmable Logic Controller (PLC) in a manufacturing company. The system was connected to the company’s production cycle, resulting in reduced scrap rates and faster training of new operators. The study will be used as a source for future research on the potential for connecting virtual instruction manuals to production systems. One aspect of the implementation included the use of a monitor at the workplace to display the virtual instruction manuals.

The paper [40] discusses the use of digital twins to support decision-making in workstation design and logistics operations. The digital twin was developed using Unity and simulation software IPS and was tested in a production logistics testbed that included physical devices, an IoT infrastructure, and simulation software. The testbed included the following items in the physical environment: collaborative robot, automated guided vehicle (AGV), vision system, virtual reality (VR) equipment, real-time locating system sensors, kitting material including parts and boxes, transportable racks, door, elevator, and connectivity infrastructure including routers. The results show that the granularity of the underlying simulation model and the level of reality in the digital twin must be adjusted according to the specific context. The production logistics testbed allows for the development and testing of supply chain transparency and smart factory-internal production capabilities. The tests and experiments demonstrate how a gradual transition to digitised logistics can take place, allowing companies to experiment with different combinations of technology to find the right level of automation for their needs.

New architecture for digital twins that uses local product and resource twins to create higher level system twins is proposed in [41]. The architecture includes a physical entity, a communication layer using Arduino Mega 2560, and a virtual space. The physical entity is a Fischertechnik model factory with sensors that capture real-time data on the factory’s resources. The communication layer uses the Arduino Mega 2560 microprocessor to transmit data between the physical entity and the virtual space, where a real-time database creates a digital shadow. A main learning outcome of the study was that the Programmable Logic Controller (PLC) is an essential part of a digital twin implementation, which currently receives insufficient research attention. Future research should focus on the link between PLCs and digital twins, including the use of wireless networks.

This study [42] introduced the use of digital twin technology in the commercial production phase of an automotive production line. The study found that the use of digital twin technology led to a 6.01% increase in production line efficiency and an 87.56% reduction in downtime. The study used various sensors and software, including the Unity platform, to collect, transmit, store, and evaluate data. The results of the study demonstrate the potential of digital twin technology in the automotive production industry and the potential for further improvements through the use of artificial intelligence techniques such as machine learning and deep learning.

The aim of this work [43] is to address the lack of experimental equipment in universities and improve existing PLC teaching methods by using 3D modelling tools and creating interactive animations of classic PLC programming cases. The resulting Windows application is developed using the Unity engine and serves as a simulation teaching platform. The platform allows students to use PLC to impose real-time control strategies on objects in a virtual environment, improving their PLC programming abilities and innovation consciousness. The platform uses Siemens S7-300 series PLC for testing and communication is achieved through the use of PROFINET and TCP/IP protocols.

Article [44] proposes a digital twin solution for the optimisation of production lines. The solution involves three steps: creating a simulation model to imitate real-world behaviour, applying automation engineering to communicate between the real world and the virtual model, and integrating simulation and automation to realise the digital twin. A programmable logic controller (PLC) code is used as a communication language to connect the real world and the simulation model. The paper presents a case study that uses this approach to optimise the routing of automated guided vehicles (AGVs) in a matrix production line for a battery assembly plant. The digital twin solution is used to validate the layout and optimise material flow, and to evaluate the effectiveness of automation technologies without disrupting the plant’s output.

### 2.4. Non Manufacturing Application of Digital Twin

In this study [45], a three-dimensional virtual traffic lights experimental model based on Unity is designed, which realises the function of the virtual PLC (PLCSIM) and Unity three-dimensional virtual model in real time communication. Using the C# language, which is supported by Unity, to realise the data transmission, this three-dimensional virtual experiment model is more realistic to show the state of the reality, which provides a feasible method for the development of the three-dimensional virtual experiment, saves the investment, and can be used as an experimental example.

The paper [45] presents a virtual PLC experiment scheme using Siemens virtual PLC and a three-dimensional virtual experiment model based on Unity. The scheme aims to provide a cost-effective and flexible method for PLC experiment. It uses Siemens PLC programming software and PLC simulation software to provide control signals, and Unity to create a three-dimensional virtual experiment model. The virtual PLC and Unity model can communicate in real-time to show the state of the control signals. An example of a three-dimensional virtual traffic light experimental model is given to demonstrate the feasibility of the scheme.

A novel intelligent vehicle behaviour analysis framework based on a digital twin is proposed in [46]. The framework uses deep learning to detect vehicles, and implements Kalman filtering and feature matching to track them. The vehicles are then mapped to a digital twin virtual scene built in the Unity game engine, where their behaviour is tested according to customised detection conditions. The stored behaviour data can be used to reconstruct the scene for secondary analysis. The framework performs well in detecting a range of abnormal behaviours, but still has limitations in the case of occlusion, distance from the camera, or small sample sets of vehicle types. Future research will focus on expanding the dataset and improving detection accuracy in these cases.

A case study of personalised adaptive cruise control (P-ACC) is conducted in [47] to showcase the effectiveness of a proposed digital twin simulation, where the ACC system can be designed to satisfy each driver’s preference with the help of cloud computing. The simulation architecture consists of two layers: the lower layer is the physical world, which contains the real-world objects being simulated; and the upper layer is the digital world, which has three sub-layers: Unity game objects, a Unity scripting application programmable interface (API), and external tools. The study shows the effectiveness of the proposed digital twin simulation in satisfying each driver’s preference with the help of cloud computing. In the next steps of the study, the digital twin simulation will be integrated with the Robot Operating System (ROS) and realistic wireless communication models to further improve the fidelity of the simulation.

This thesis [48] presents the development of a digital twin, a real-time simulation of a 3D compensated gangway located on a service operation vessel for wind farms. The simulation is implemented in the Unity game engine and interfaces with a control system. Testing shows that the simulation, which runs on a separate thread with a 5 µs step time and 10ms latency, is suitable for use as a hardware-in-the-loop test setup. The simulation enables testing and implementation of a 3D compensation control system with acceptable accuracy, as well as testing and tuning of a pressure feedback system. The use of a game engine to simulate a hydraulic system in near real-time is a novel approach that allows for the creation of realistic simulations and provides the ability to include feedback from hydraulic sensor values. The hardware-in-loop setup can be extended to other products and used for data collection to improve its validity as a digital twin.

### 2.5. Applications of Digital Twin Using Fischertechnik Education Models

Articles [49,50] present a framework for creating Intelligent Digital Twins (IDTs) in the context of the Internet of Digital Twins (IoDT) and the convergence of Industry 4.0 and Industry 5.0 paradigms. The authors propose a DT reference model aligned with Industry 4.0 and enriched with Industry 5.0 goals, and demonstrate how IDTs can be realized with the characteristics of Multi-Agent Systems (MAS). An architectural design for IDTs is presented, which coordinates marketplace-oriented production processes. The design is tested through a prototype implementation and a proof of concept using Fischertechnik Training Factory Industry 4.0.

In this paper [51] the focus is on the creation of the Digital Shadow for the Training Factory Industry 4.0, which was developed as part of a university course. The primary goal was to investigate whether the skills and knowledge obtained during the Computer Science undergraduate program were adequate for building a functional Digital Shadow of the training factory.

This bachelor thesis project [52] analyzes the Fischertechnik 4.0 Training Factory simulation model within the context of Industry 4.0. The model is complemented with a digital twin created through Siemens Tecnomatix Plant Simulation software, and an Initial and To-Be scenario are created for analysis. The C++ code used in the model allows for the introduction of different 4.0 features and devices, but due to lack of knowledge, these aspects could not be developed further. The Siemens Tecnomatix software is analyzed through the scenarios, and two manuals are created to make using both tools easier.

Author of [53] created a digital twin of a Fischertechnik factory simulation as part of his master’s thesis at Quality Automation. The twin was developed using CAD files and Siemens program CAD/CAM system NX, and meshing was defined for each element and sensor. The virtual commissioning of the digital twin was carried out with a simulated PLC. The project included a direct comparison of the hardware model and its purely virtual twin, highlighting the possibilities of virtual commissioning and the increasing importance of simulations and virtual twins in the future.

This bachelor thesis [54] compares the use of digital twins in three different phases of a product’s lifecycle: design, production, and service. The comparison focuses on user, functional, data, and physical object aspects. The results show that using digital twins across all three phases provides the most benefits to companies, despite each phase having its unique characteristics.

### 2.6. Summary of Related Works

Based on the analysis of related works, it can be stated that most of the analysed works declared in their title or texts that they work with digital twins. Based on the elaboration and explanation of the difference between the terms digital twin and digital shadow in the Introduction section, it can be stated that most of the analysed papers proposed only digital shadow (data flow was only directed from the real system to the virtual one). We confirmed the claims in the Introduction section that digital twin is often used only as “scientific” marketing, a buzzword or the authors do not have enough study on the subject. The works that can certainly be considered as digital twin in the above mentioned context are the works focused on robotic workstations that have made it possible to control a real robotic system by using its digital twin.

The goal of this paper is to present a practical method for creating digital twins, both simple and complex. To achieve this, we have designed easy-to-follow educational case studies that utilize readily available tools and techniques. The primary focus of these case studies is to establish two-way communication between the actual system and its digital twin.

Our intention is to use these case studies as a starting point for developing training courses for Industry 4.0 education. Additionally, they can be further modified to create Industry 4.0 educational materials and technical resources.

## 3. Aim of Methodology

**Figure 2:**
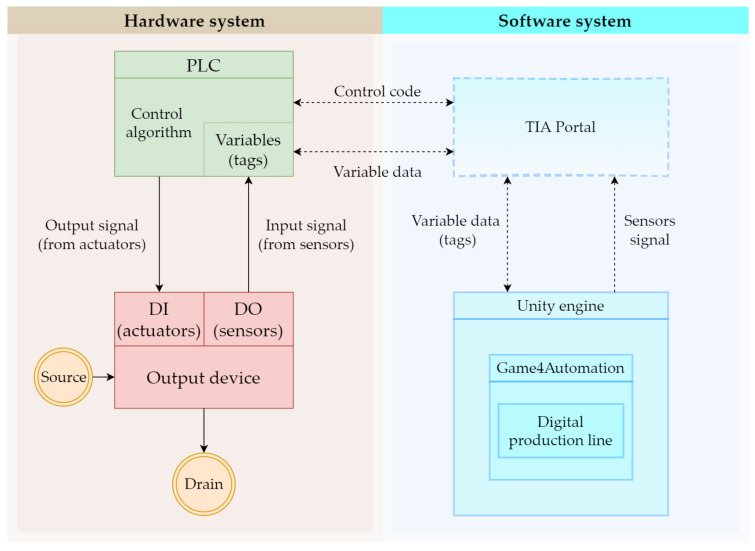
Scheme of connection between hardware and software.

Before starting the actual work, we have to know what kind of tools, either hardware or software, we will need. We start with the hardware specifications of our work. The first thing we need is a device that we are going to transfer into digital form. We have used two devices. The first is an assembly line and the second is a warehouse stacker (both made by Fischertechnik), both of which we will describe in detail in the case studies. Next, we need to control these devices. Here we have chosen a PLC device from Siemens. Specifically the S7-1200 series with a 1215 DC/DC/DC CPU. The connection will be made via an Ethernet cable. In the software part we will first work with the TIA portal program, which will serve us for communication between the computer and the PLC device and at the same time we will program the transport line and the warehouse stacker. The digitalization itself will be carried out in the Unity game engine. Unity will serve only as an environment in which we will work. The Game4Automation framework will play the main role in the digitalization. It is in this framework that we will try to replicate the real object as closely as possible. In Figure 2 we can see the interconnection of the line, the software, and their mutual dependence in two-way communication.

## 4. First Case Study: Digital Twin of an Assembly Line

The aim of our first case study is to link the physical model of the assembly line with its digital version. At the same time, we want them to interact with each other, that is, if we perform an action on the real model, then the same action is automatically performed on the digital version and vice versa. Our discrete-event system (Figure 2) should behave as follows:

- Two-way communication between the physical digital line will be ensured;
- The digital and physical lines will influence each other, i.e., an event executed on one will be manifested on the other.

In the next section, we will describe the tools we used in the development of our application. We tried to choose the tools in such a way that our work would be as efficient and clear as possible, especially when it comes to the software. We will go through the hardware components first and then move on to the software.

**Figure 3:**
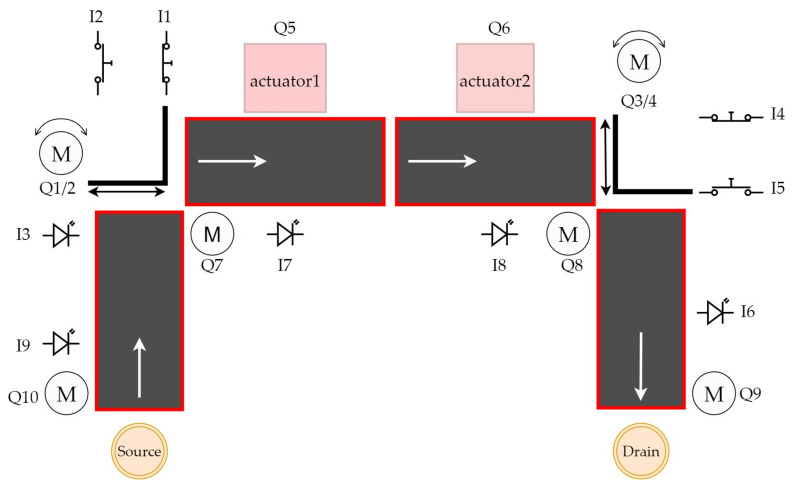
Electrical scheme of assembly line.

Our work consists of transferring a physical object into a digital environment. The physical object we have chosen to transfer is a model of a production line from Fishertechnik [55]. In the Figure 3, we can see the scheme of the assembly line. This model is a simplified version of a large production line. It consists of:

- four conveyor belts;
- five light gates;
- two sliding devices;
- two stations that simulate a drill and a milling machine.

We will go through a deeper cross-section of the line in the next subsection. We can divide the line into two types of components. The first type is input devices, i.c., devices that receive stimuli from the external environment and send signals based on these stimuli. The signals are received by the second type of components—the output devices. These evaluate the signal they have received and carry out their operation on that basis. The input devices include:

- a light gate;
- a slider position sensor.

A light gate is a photosensitive device that emits light on one side and receives it on the other. If the light beam is interrupted, the gateway sends out a signal. Another input device is a slider position sensor. This input device senses the position of the slider, which is a device that moves an object from one conveyor belt to the next, in places where the conveyor belt does not have reach. This sensor is a button that reacts when the slider is directly above it, and therefore presses it. Output devices include:

- a conveyor belt;
- a slider;
- drilling and milling station.

**Figure 4:**
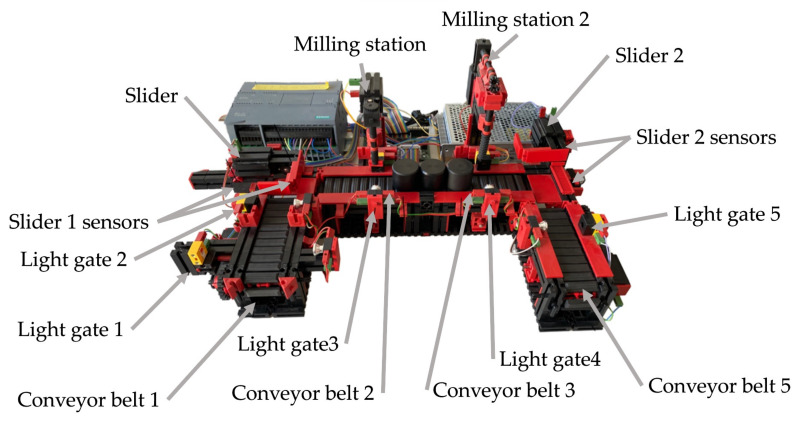
Overview of the line.

The conveyor belt and slider start moving in a predetermined direction when a signal is received, in the case of the stations, this is only a simulation. In the Figure 4, we can see the whole production line.

### 4.1. Overview of the Line

**Figure 5:**
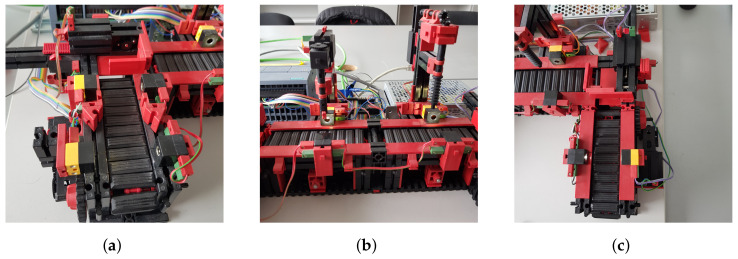
Three parts of the production line. (**a**) Beginning of the line; (**b**) Drilling and milling station; (**c**) End part of the line.

In the previous section, we described what devices the line contains and roughly described how they work. In this part of the article we will describe all the components, including the line itself, in more detail. The first thing to mention is that the line can only be controlled via a 24V PLC device. The PLC device that we used to control the line has the designation S7-1200 and belongs to the simpler PLCs by Siemens. The whole line is U-shaped (Figure 4). At the beginning we have a light gate waiting for the goods, followed by a conveyor belt, another light gate, a slider (Figure 5a), a milling and drilling station (Figure 5b) and again a slider, a gate and a conveyor belt (Figure 5c). All the equipment that performs the movement is driven by DC motors. There is also a panel on the model which contains the circuit boards on which the relays are located. These ensure that the motors can change direction. In addition, all inputs and outputs can be connected to a jack connector or series connected terminals.

#### 4.1.1. Light Gate

The light gate is made up of two parts: a photo transistor and an LED bulb. Thanks to this, the gateway produces an input signal that can drive different parts of the line. The dimensions of one light transistor are 15 × 15 × 7.5 mm with a weight of 1.7 g. In terms of functionality, the gateway receives the light that it transmits from one side to the other. If its light beam is interrupted, then it evaluates the signal as FALSE, and vice versa. If the light is uninterrupted, then the signal is evaluated as TRUE. With this function, it is possible to determine the position of the goods on the line or to move any output part of the line [56].

#### 4.1.2. Mini Switch

Another input device is a mini switch. This device determines the position of the slider according to whether it is pushed. Mini switches are located at either end of the slider and whichever is pressed determines the position of the slider. In total, the line contains four. The dimensions of the switch are 30 × 15 × 7.5 mm with a weight of 3.4 g. The slider itself is located in places that the belt cannot reach and its role therefore, is to transport material from one belt to the next [56].

### 4.2. Unity Game Engine

**Figure 6:**
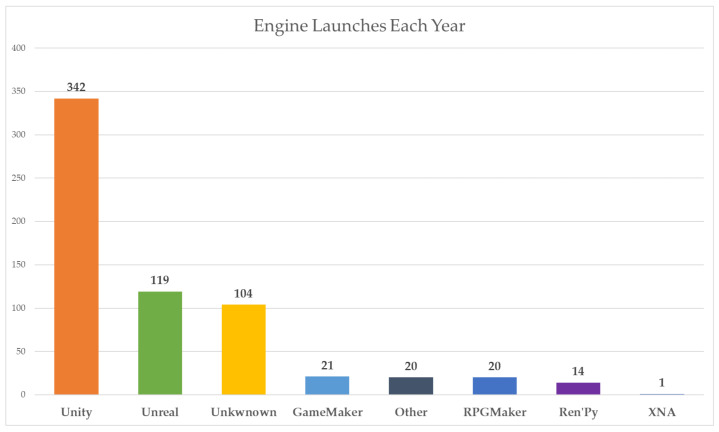
Annual launches of 3D engines on Steam platform [57].

We used the Unity game engine to build the line model itself. This engine is used to create 2D and 3D games across different platforms. The decision to use the Unity engine was made for several reasons. The first reason was our previous experience with Unity, so it seemed like a good programme to try and build our work in. Another reason was its features as a game engine, where various features for games, such as physics and the ability to create 3D objects, are pre-prepared. Before we discovered the Game4automation framework, we assumed that we would have to model a simple line model directly in Unity. A large library called the Unity Asset Store helped us a lot during our work. It makes adding frameworks and other components very easy. In Figure 6, we can see a big difference in the use of the Unity engine compared to the competition.

#### Game4Automation

The original solution to our problem was to try a Node-Red middleware to link the hardware and software and work with the data that the line sends, then we would have to store, process and overwrite those data every time there was a change on the physical line. Since this procedure seemed complicated, we decided to look for other solutions. While looking for our options, we discovered the Game4Automation framework, which is available directly in the Unity Asset Store and is therefore fully compatible with Unity. This type of programme solves all the problems related to the individual interfaces between hardware and software, and also solves the visualisation of the digital twin thanks to a library of models. Basic components include conveyor belts, sensors, or goods. However, the framework also offers an extended downloadable library directly in the Unity Asset Store, which contains components from various companies. Each version of the framework is paid for. We worked with the basic and therefore cheapest version. This version provides connectivity to all types of PLC hardware by Siemens (S7-300, S7-400, S7-1200, S7-1500). Communication is provided by the S7 TCP-IP protocol, but higher versions also offer other types, such as OPC UA or Modbus. The S7 TCP-IP interface is based on Snap7 Ethernet communication. This is used for Ethernet communication with PLC systems [58]. To give even a beginner an idea of how the individual components work, the framework offers simulated scenes where these components are used. Of course, if the basic functions are not enough for someone, the framework provides the possibility of reprogramming the scripts.

### 4.3. Preparation of Tools

In this section, we will go through configuring the various software and hardware tools. We will cover individual interfaces, starting and programming a line or installing a framework. Gradually it was necessary to:

- connect the computer with the Line Model;
- program the line;
- install and run the Game4automation framework;
- link between Game4Automation and TIA portal;
- export tags.

#### 4.3.1. Connection between PLC and Computer

**Figure 7:**
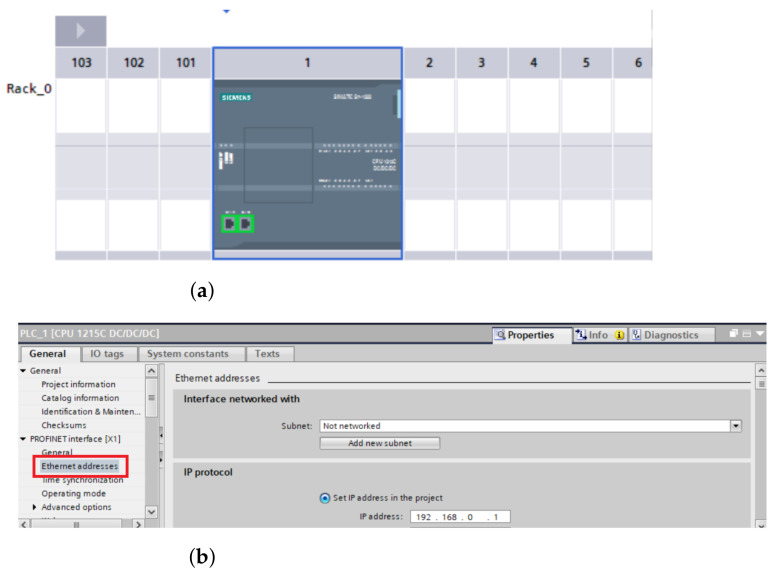

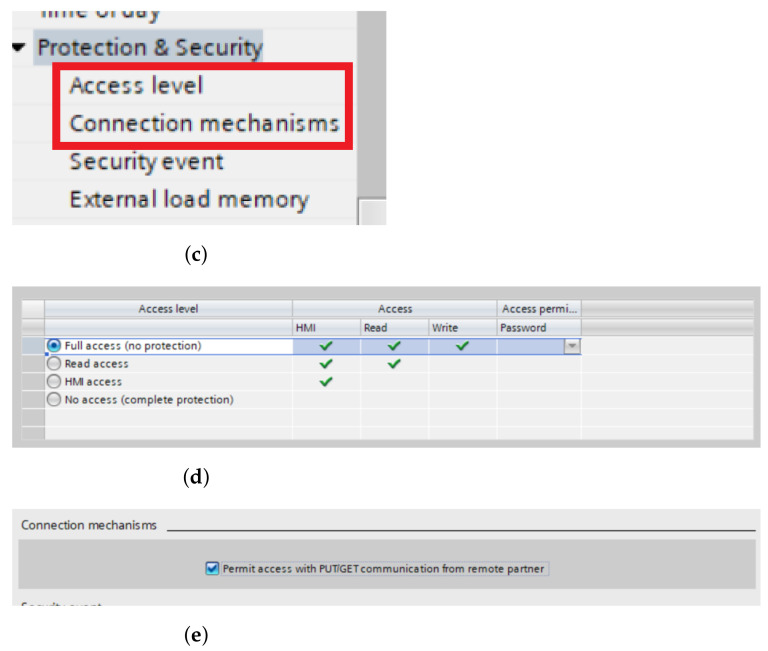
Device and networks settings. (**a**) PLC device picture; (**b**) Adding PLC IP adress; (**c**) Protection and security; (**d**) Access level; (**e**) Remote partner permission check.

The first step that must be taken is to connect the *PLC* device to our computer. As far as the physical side is concerned, this is provided by the Ethernet cable that goes directly from the computer to the *PLC* device. On the software side, we used the *TIA portal*. If the cable is properly connected and it is not damaged, we can locate our *PLC* device in the TIA portal programme if we know the IP address of our PLC, then we can write it directly (Figure 7b). However, before the actual procedure, it is necessary to have the device offline. Only then we can proceed to connect the device. In our case, we are using the *S1215 DC/DC/DC PLC* device. After clicking on the device and network options, the PLC image will appear (Figure 7a), which we will click on twice. Next, the PLC device settings will open. Since we also need to have proper access and the ability to overwrite the content in our *TIA portal* via external devices, we also need to check the accessibility (Figure 7d) and check the PUT and GET options for other devices (Figure 7e). These are located in the “*protection*” and “*security*” section (Figure 7c). The “*access level*” option should be set to “*full access*” from the start. If this is not the case then this needs to be set. Finally, the section in “*Connection mechanism*” should be enabled for PUT/GET communication from remote device. At this point, we should have full access to our device and also have it fully connected.

#### 4.3.2. Starting the Line

The next task is to run or programme our production line model. For us, this means that the goods arrive from one side of the line to the other, stopping at the drilling and milling station. In programming we have used four types of tags, namely:

- physical inputs;
- physical outputs;
- virtual inputs;
- auxiliary variables.

**Figure 8:**
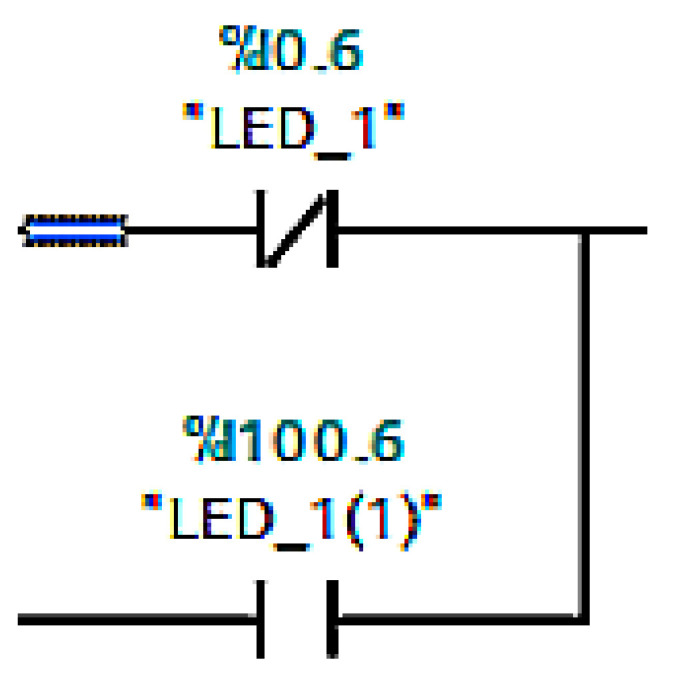
Example of using virtual input.

Physical inputs include light sensors and mini switches and outputs include conveyor belts or sliders. The problem arose when we wanted to override the physical inputs directly in *Unity*. This task is not possible, so it was necessary to use virtual inputs. Virtual inputs are created by adding the value 100 to the current value, for example, %I0.6 is %I100.6 (Figure 8). Finally, we used auxiliary variables to better determine the position of goods on the line. An example is the variable *ZLED1DoLED2* (in translation from LED1 to LED2), which is set as TRUE when the goods cross the first led and FALSE when the slider is in the back position.

#### 4.3.3. Home Screen

**Figure 9:**
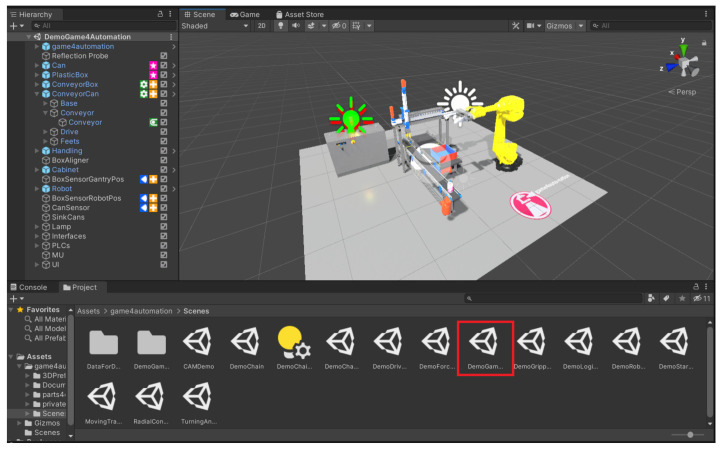
Demo scenes.

**Figure 10:**
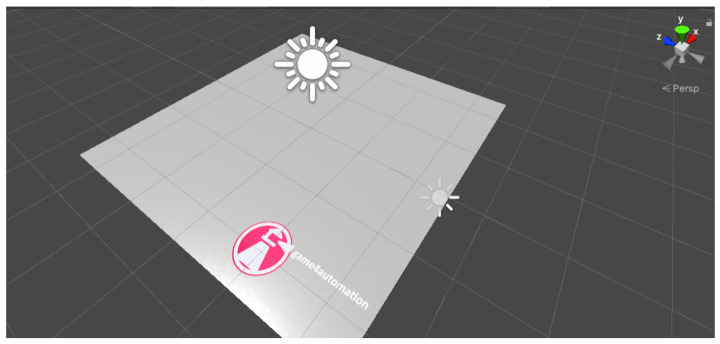
New Game4Automation scene.

When we first open the Game4Automation framework, we are presented with a dialogue window where we have a variety of options to choose from, such as documentation, YouTube video tutorials, a link to the forum, and more. After closing this window, we can see an initial demo scene that shows a working model (Figure 9). In the left panel we can see what components are in the scene and in the bottom panel we can see the structure of the whole framework, from prefabs to scenes or scripts. For the framework to work properly, it is not enough to create a new scene in Unity alone; we need to create a new scene directly in the framework. We do this as follows. On the top bar that starts with the File button, press the Game4automation button. In the menu that opens, selecting “Create new Game4automation” scene will create a new scene (Figure 10).

#### 4.3.4. Inputs and Outputs

**Figure 11:**
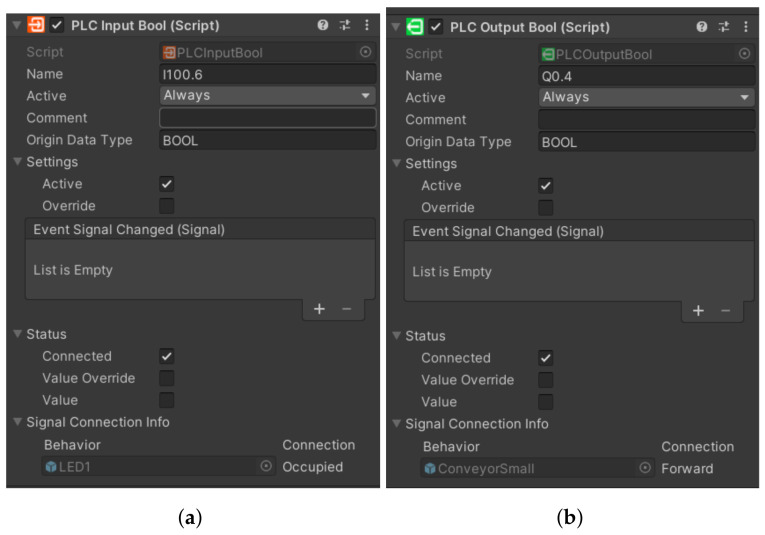
Input and output interface. (**a**) Input; (**b**) Output.

**Figure 12:**
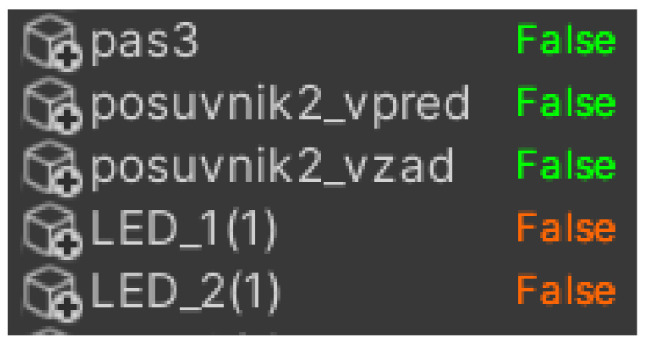
Input and output components.

In the same way that a production line has its input and output devices, a framework also has inputs and outputs that serve as a virtual representation for the physical ones. We defined the first virtual representations in the TIA portal programme, where we stored them with values of 0, for example, q0.0 for output device or I1.0 for input device. However, when we want to overwrite these values, we must also store them with values of 100. This means that we have to add the value 100 to the values before the dot, i.e., Q100.0 for Q0.0. Otherwise the programme would not work, since we cannot overwrite physical variables directly. If we store them with values of 100, we will be assured of real-time overwriting. Directly in the framework, inputs are represented in orange (Figure 11a) and outputs (Figure 11b) in green. Apart from that they are not very different. Their structure is very similar- there is a label of the input/output, an activity condition (in our case they are active all the time), a data type or settings, where we have two choices. The first option is whether we want our variable to be active. The second is whether we want to overwrite the value manually in Unity. In the status option we can see if the variable is connected: its value (in case of BOOL it is TRUE and FALSE) and likewise if it is overwritten. In the last field, signal connection info, we can see if the variable is connected to any sensor or other component. Since we cannot add inputs and outputs directly to the simulation environment, we have to connect them to the individual components that correspond to their roles. For example, we connect the light gate to the sensors, and similarly, we connect the output from the conveyor belt that sends a signal that motion is in progress to the script for the virtual belt motion in the Figure 12, we can see the input and output devices.

#### 4.3.5. Connections between Unity and TIA Portal

**Figure 13:**
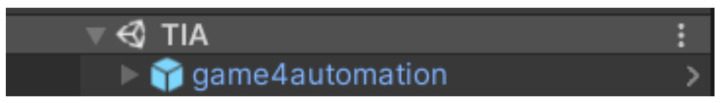
Game4Automation icon.

**Figure 14:**
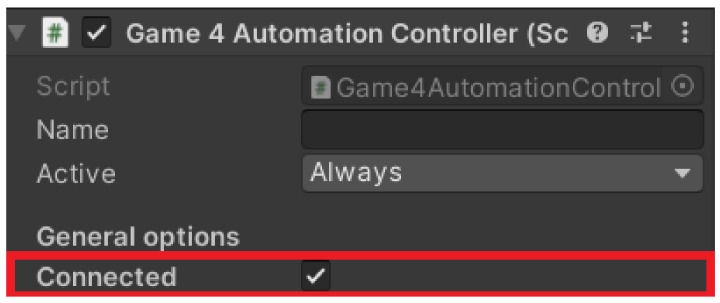
Game4Automation connected option.

**Figure 15:**
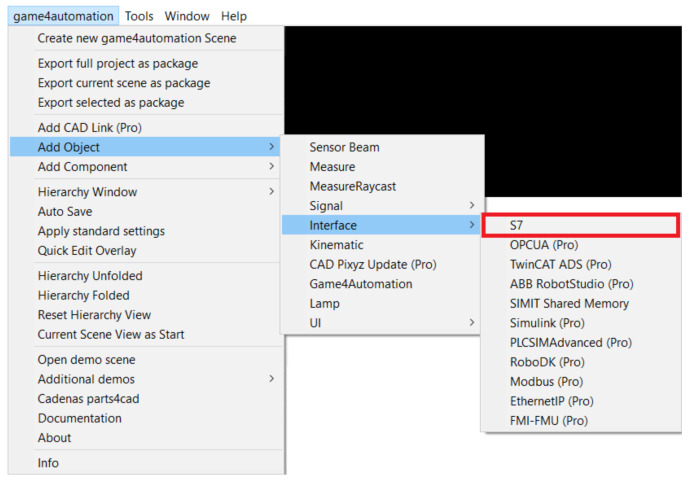
PLC device S7-1200 used by us.

Before connecting, we clicked on the Game4 Automation icon in the left panel (Figure 13) and selected the connected option (Figure 14). This ensured we were automatically connected whenever the simulation was turned on. Next, we had to set up the Connection directly in the framework. With our version we only had one option, via TCP–IP, and this was set as follows. Again, we returned to the top bar to the Game4 Automation option and proceeded as follows: Add object -> Interface -> S7. With this we have added the user interface for the S7 connection (Figure 15). In the right panel we can see the interface options we used. The first one we needed to set was the IP address of our device. In our case it was 192.168.235.88. Now it was necessary to import the variables that are used in the TIA portal into our project in Unity.

#### 4.3.6. Tags Exporting

**Figure 16:**
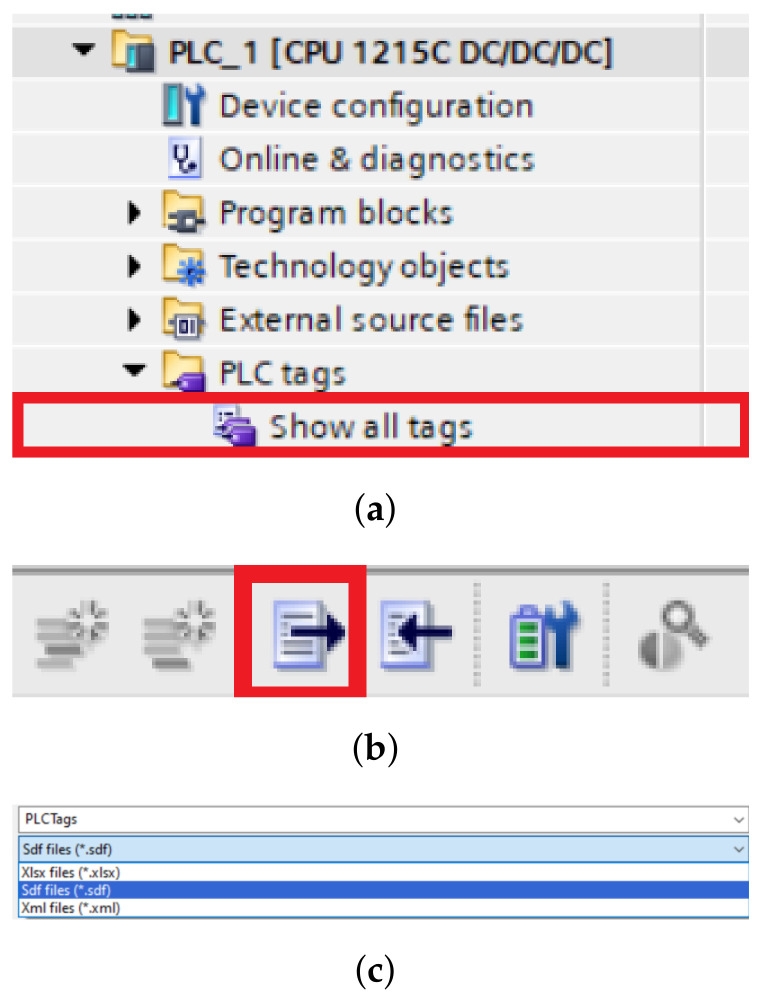
(**a**) Section with all tags; (**b**) Saving in sdf format; (**c**) Saving as sdf.

The variables are imported as follows. We open a table of all the tags (Figure 16a) we defined when programming in the TIA portal, then export them to a table that has the extension .sdf and we can move to Unity (Figure 16b,c). In Unity we need to import our variable table, and we do that as follows: First we need to click on the Select symbol table button and select the table we have saved, then we click import symbol table and if we have selected the correct table, we should see all the variables we have saved in the left panel. To check the connection, we need to click on the check connection button. If the device is properly connected, the variables will turn from grey to either green or orange, depending on whether it is an input or output. In our work, a problem occurred when choosing between the two options. The first option was the *Only Write To PLC changed signal* and the second was the *Area Read Write Mode*. The disadvantage of the first option is that it only works for a limited number of variables, so when we had more of them and tried to turn on the programme, although it turned on, it was unable to connect to the PLC device. The second option, as the name suggests, sends a signal to multiple variables at once depending on the memory we select. The manufacturer itself indicates in the documentation that this option was added because of projects that contain a larger number of variables. In the end, we opted for the first option and reduced the number of variables that the digital twin will use. In our testing, it sometimes happened that, despite the connection we addressed by following the procedure, the framework did not want to start the line even though it showed that it was connected. We eventually fixed this bug by creating a new project in the TIA portal programme, which only contained a very simple script to run the line through the light gateway signal. After uploading and running the script, and then re-running the original script, everything was working properly for us.

#### 4.3.7. Components

The components are used to visualise the real device so that it is as true to its model as possible. The framework itself contains basic generic components that allow us to build common, less complex devices such as our line. However, if one needs a larger number of components, then one would need to download the extended component database from the Unity Asset Store anyway, or combine components to create their own, as we did when creating the slider component. The components themselves can be divided into two types. The first type are non-moving components, such as a box or a can, which do not move without external influence; the second type are moving components, such as a conveyor belt, a piston, or different types of machines. We will describe the importance of the individual components, especially in the case of our work, in the next section.

#### 4.3.8. Static Technical Components

In our work we used two static components, namely a box and a can. The box serves, similarly as in reality, to transfer the material if necessary, but this transfer must already be provided by a dynamic component, such as a belt or a crane. The can represents the material that is loaded onto the line. The difference between an ordinary component and a can is that it contains a script that allows it to be cloned whenever the previous can has travelled a specified distance from the starting point.

**Figure 17:**
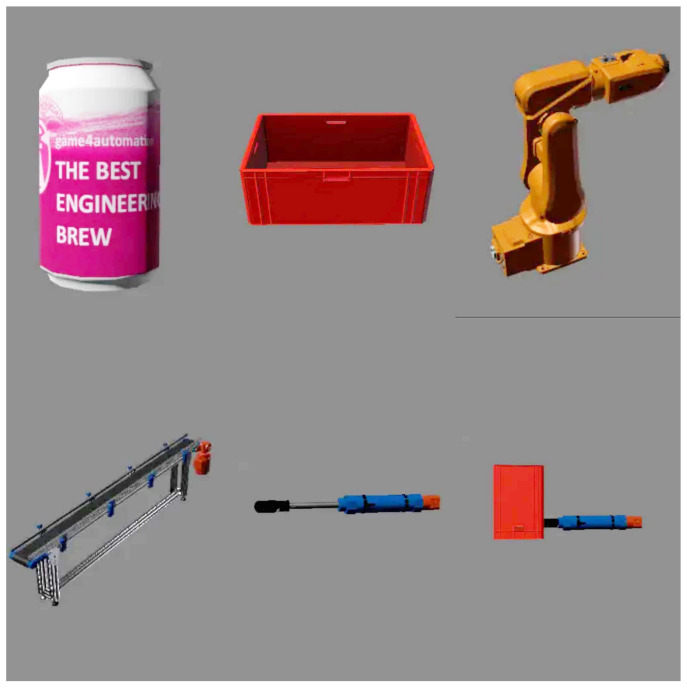
From the upper left corner are the can, box, robot, conveyor belt, piston, and slider.

These components in our work include a conveyor belt, a piston or slider that we constructed by connecting the piston and the box, and robots that simulate a drilling and milling station. Different types of scripts are used to move them, which we select according to the type of motion we need from the component. For us, using a particularly basic motion script called drive simple was sufficient. All mentioned components can be seen on Figure 17.

#### 4.3.9. Script Drive Simple

**Figure 18:**
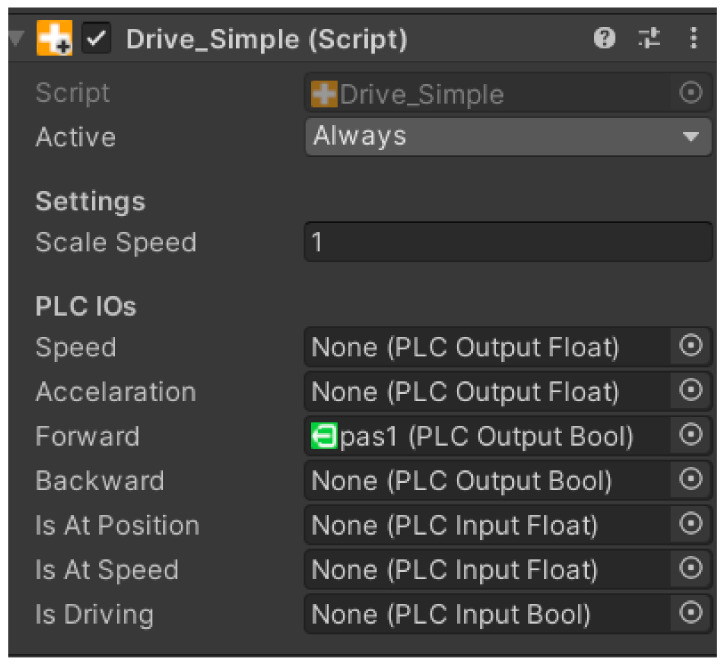
Script drive simple.

The script ensures easy forward and backward movement of the component. The logic of how the script should work is contained in a table that is displayed after the script is inserted into the component. The logic works based on signals from the PLC device. Below we can see what an overview of the drive simple script logic looks like (Figure 18).

- Speed—initialisation based on PLC output Float;
- Acceleration—initialisation based on PLC output Float;
- Forward—initialisation based on PLC output Bool;
- Backward—initialisation based on PLC output Bool;
- Is At Position—initialisation based on PLC input Float;
- Is At Speed—initialisation based on PLC input Float;
- Is Driving—initialisation based on PLC input Bool.

Each component has specific tasks. Speed determines the speed of the device, Acceleration determines the acceleration, and Forward and Backward determine which direction the device should start moving after receiving a specific signal. We can use the last three options for logical tasks, depending on what the component is supposed to do in a given situation. We have the ability to override all scripts.

#### 4.3.10. Sensors

**Figure 19:**
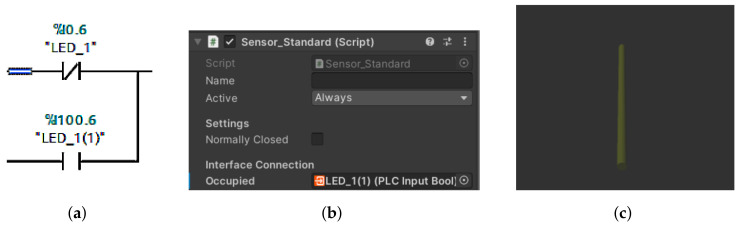
Different interpretations of input device. (**a**) Logic gate OR; (**b**) Sensor inputs; (**c**) Object of sensor.

The sensor is represented in the framework as a yellow beam. Thanks to the sensor (Figure 19b,c), we can virtually represent the input devices from the real model. The sensor has two roles in our work. The first is to send signals directly to the digital twin, in other words it acts as a light gate in the real model. The second role is to utilise the signals to move the real machine. In order for the sensor to work properly, it must be placed in the necessary location and we must assign it the variable we want it to override. The truth value of the sensor is exactly opposite to that of the light gate. If nothing crosses it, then the signal is FALSE and, vice versa, if material crosses it, then it sends a TRUE signal. The sensors must also be correctly included in the code in the TIA portal. This means creating a logical OR gate (Figure 19a). This gate will receive the signal from the sensor and at the same time from the light gate and will trigger the conveyor belt whenever at least one branch contains a TRUE signal. Once the tools are properly prepared, the last task comes next, in which our entire project is programmed and fabricated.

#### 4.3.11. Virtual Line Assembly

**Figure 20:**
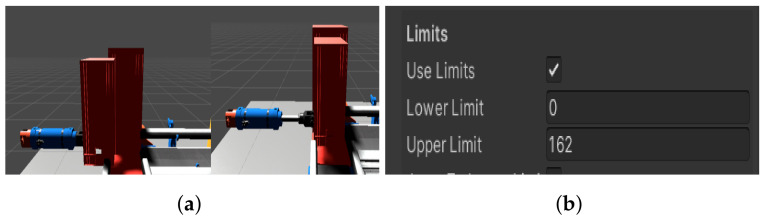
Virtual Slider with limiter. (**a**) Virtual slider; (**b**) Limiter.

While the real assembly line has to be programmed, the virtual one has to be assembled. The production line is a simple linear device, in other words the goods go directly from point A to point B. It is generic anyway, so we only needed the basic parts. The only part we created was a slider (Figure 20a), as we could not find anything similar in the basic package. In addition, in our work we used conveyor belts, sensors, a can to simulate the goods, a box as a platform between the belts, and two machines to simulate a drilling and milling station. Furthermore, we set constraints on the movement of the piston so that it moved only in a limited way and acted plausibly (Figure 20b). We then fitted the components to match the real line. Compared to the real line, we omitted the mini switches to determine the position of the slider and solved the problem through the signals according to which our digital slider extends and retracts. These signals are sent to us by the real slider. Once all the components are correctly stored and wired, we move to the last stage, which is wiring the inputs and outputs into the logic of each component.

#### 4.3.12. Simulation

**Figure 21:**
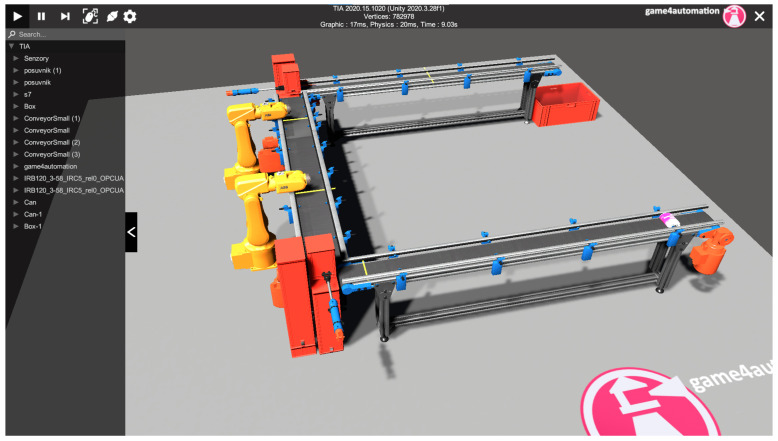
Digital twin.

Once all the components of the virtual and real aspects of the task were running correctly, we ran the simulation. If everything is wired correctly, then the twins work bilaterally, i.e., the physical twin can control the virtual twin and vice versa. In the first tests, we investigated whether the virtual twin can control the physical twin. The only difference was the speed of the goods transported, or the length of the conveyor belts. Fortunately, the framework allows changing the length of the components or accelerating the conveyor belt. In the same way, in the TIA portal environment it is possible to change the start length of the real conveyor belt. When tested in the opposite direction, no problems were encountered as each virtual component moves as long as its physical counterpart does. Something to watch out for during work is the restart of the CPU of the PLC device after each interruption of an incomplete simulation. Otherwise, the next simulation did not work properly. In the Figure 21, we can see the finished virtual line. In Figure 2 we can see the communication scheme between the physical layer and the softer layer. At the same time, the figure shows us how the lines influence each other.

In this state, the digital twin allows the virtual line to influence the real one and vice versa. However, this study did not address the synchronization of events in the virtual and real line. Event synchronization will be a continuation of this work, where 3D CAD models of the real line will be used. Synchronization will also allow predictive maintenance elements, as, if events in the virtual and real lines do not occur simultaneously, it will be possible to conclude that an error has occurred on the real line, for example, a slowdown of the conveyor belt due to a decrease in motor performance.

## 5. Case Study n.2: Digital Twin of a Warehouse Stacker

**Figure 22:**
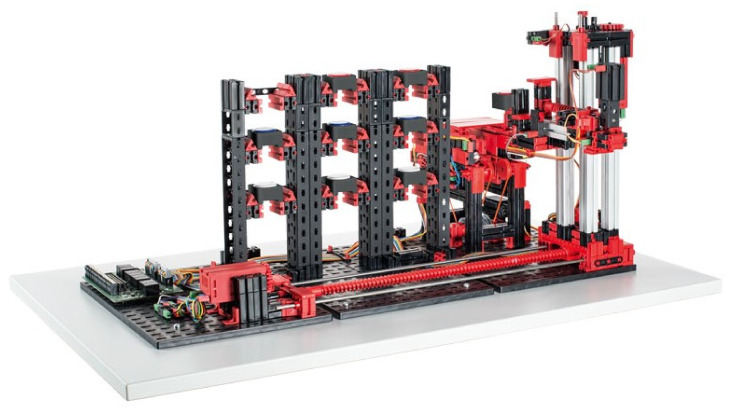
Warehouse stacker.

In the second case study, we aimed at a more complex device, which was a warehouse stacker (Figure 22). In this part we worked deeper with the framework than in the first part, which means that most of the simulation is carried out in Unity. Since it is a more complex device we use the open scripts offered by the framework to programme it.

### 5.1. Warehouse Stacker

The second case study deals with the simulation of a warehouse stacker from Fischertechnik. Warehouse stacker is a sorting robot with a conveyor belt and a shelf stacker for storing and removal of carriers. Now we will describe the functionality and parts of the model.

#### 5.1.1. Warehouse

A warehouse is an area for storing and retrieving goods. In most cases, such warehouses are designed as pallet rack warehouse systems. The warehouse has nine places—three rows and three floors. Per warehouse one rack of goods is stored in one warehouse position. Goods are often stored dynamically. Between positions and the goods, there is no fixed arrangement, so the stock goods are placed on any available space in the warehouse. Another stacking strategy may also be used, where the warehouse is divided into three zones according to the type of goods. As a result, the stacker system places the goods according to their type in the nearest empty space. The model contains two DC engines with encoder and two DC engines without encoder. The engines are used to move the conveyor and stacker.

#### 5.1.2. Transport of Goods

The goods enter the warehouse via a conveyor belt. The conveyor belt contains two light gates at the beginning and at the end of the belt. In the middle there is an IR sensor that senses the value—the colour of the goods in the carrier. The colour sensor records the differences between light and dark and interprets them. Reflections often occur on the edges of the goods carriers, so it is necessary to remove them, to avoid misinterpretations. When the carrier arrives at the end of the conveyor belt, the light signal is interrupted and the conveyor belt stops. At this point, the robot arm extends and picks up the load carrier.

#### 5.1.3. Carrier

**Figure 23:**
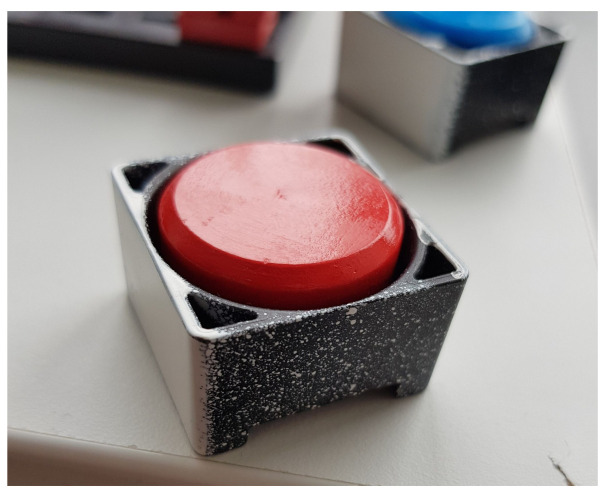
Workpiece in carrier.

The carrier has a quadrangular shape (Figure 23). One workpiece can be placed on the carrier. The workpiece has a cylindrical shape, which is distinguished by colour. There are three colour types—blue, white, and red. The side walls of the carriers must be whitewashed to avoid misinterpretations when scanning the colour of the goods.

#### 5.1.4. Stacker

**Figure 24:**
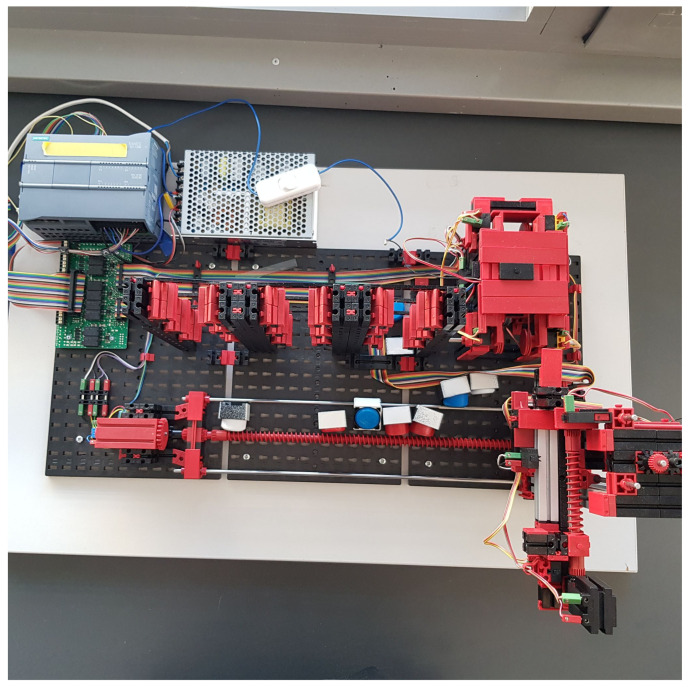
Top down view.

The stacker consists of a feeder-arm and a stand. The feeding arm is used for the transfer of carriers. The movement of the arm is vertical and horizontal (in/out). The whole rack of the stacker moves horizontally, to the left towards the pallet racks and to the right towards the conveyor belt. The stacker can perform several movements at the same time, e.g., retracting the feeder arm while approaching the warehouse. The horizontal movement of the stacker has calculated positions for the storage racks and the conveyor belt. The vertical arm movement also has calculated positions to allow the arm to insert correctly and remove the carrier in the warehouse and conveyor. The positions must be exact to avoid dropping of the carrier with the goods or poor stowage in the warehouse. In the Figure 24, one can see a top view of the whole device.

#### 5.1.5. IR Trail Sensor

The IR trail sensor is a sensor designed for colour recognition. The IR trail sensor is a digital infrared sensor to identify a black trail on a white background at a distance of 5 to 30 mm. It consists of two transmitting and two receiving elements. The captured value can be converted into specific colours (blue, red, and white) in the software programme [59].

### 5.2. Simulation Model

In this section we will describe how we built a warehouse model in the Game4Automation framework.

#### 5.2.1. Input Conveyor

**Figure 25:**
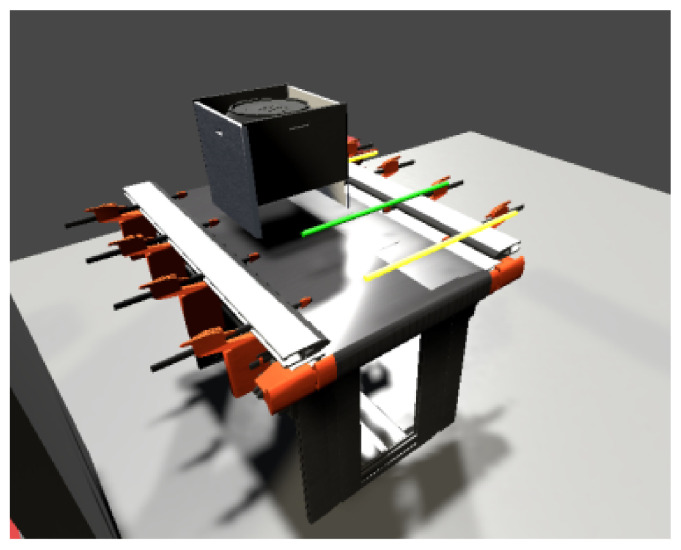
Input conveyor.

We will use an object from Game4Automation as the conveyor belt model (Figure 25). The conveyor consists of legs, a structure, and a conveyor belt. This object will serve as a starting position of the carrier. We add two sensors to the conveyor, which are located at the start and at the the end of the belt. We will simulate the IR sensor for colour sensing by using another sensor that is placed approximately in the middle of the conveyor belt.

#### 5.2.2. Carrier and Workpiece

**Figure 26:**
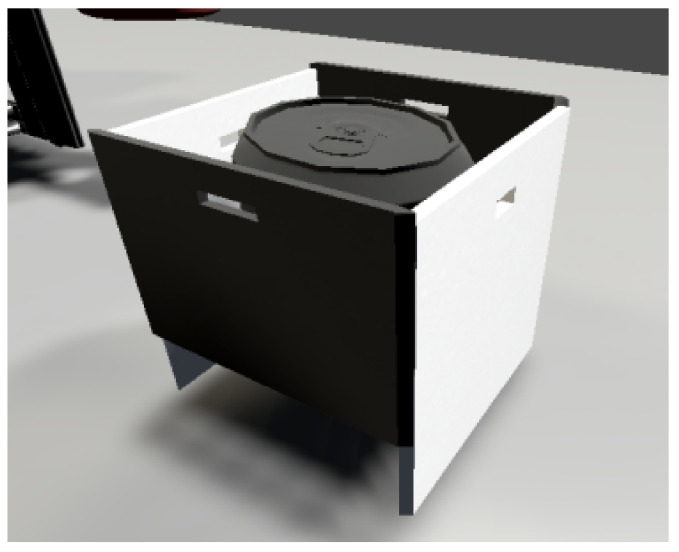
Workpiece in carrier.

We use a Box object as the carrier (Figure 26), which we modify slightly to represent the carrier according to the real template. We adjust the colours of the carrier to match the template. The white walls are on the sides so that the IR sensor can better capture the sensed colour value. The workpiece is cylindrical in shape and is placed on the carrier. We used a Can object and visually adjusted it to match the real template. When a new carrier is placed on the conveyor belt, the workpiece is always connected to the carrier.

#### 5.2.3. Stacker

**Figure 27:**
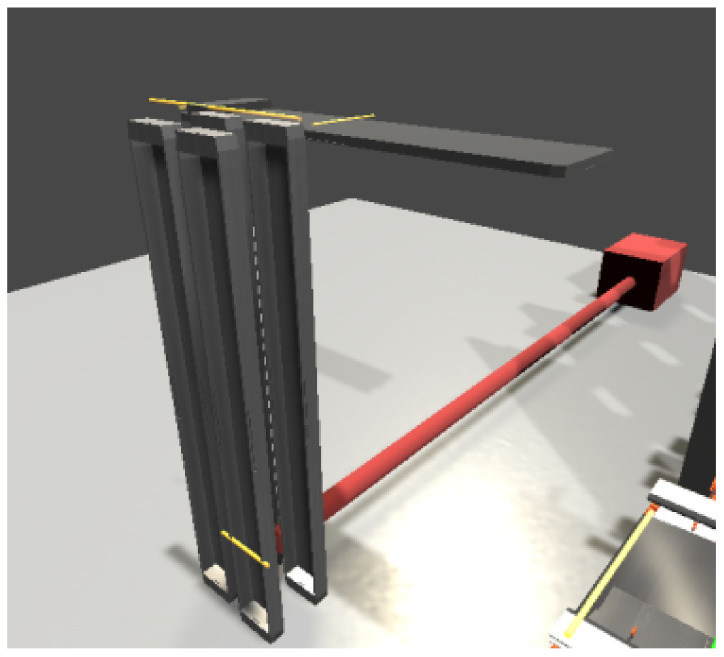
Extendable arm.

The stacker model is created using 3D objects directly in the Unity environment. By combining several objects, a simulation model of a stacker is created, consisting of four standing panels and an extendable arm (Figure 27). The switches on the stacker will be simulated via signal beams, which are part of Game4Automation.

#### 5.2.4. Warehouse

**Figure 28:**
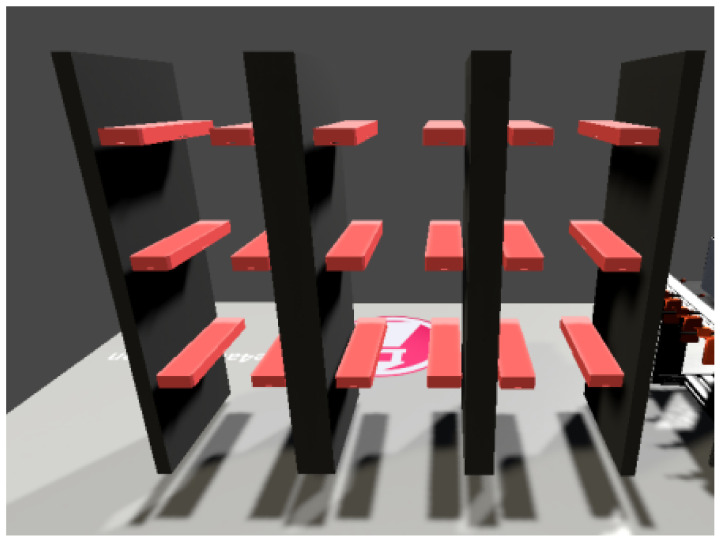
Warehouse in Unity.

Figure 28 shows a warehouse composed of ordinary Unity editor blocks. It is composed of three columns and three rows, depending on the model. The height and spacing of each position is the same as in the real stacker. For the digital twin, such a layout is necessary, i.e., all positions and coordinates must match the model.

#### 5.2.5. Switches and Sensors

We use a signal beam to simulate the switches and all sensors. We can choose which object is crossed by such a beam, for example, so that on a conveyor the sensor only reacts with the workpiece carrier. With a light beam we can set the colour (for better visualisation) and assign a PLC signal.

### 5.3. Functionality

**Figure 29:**
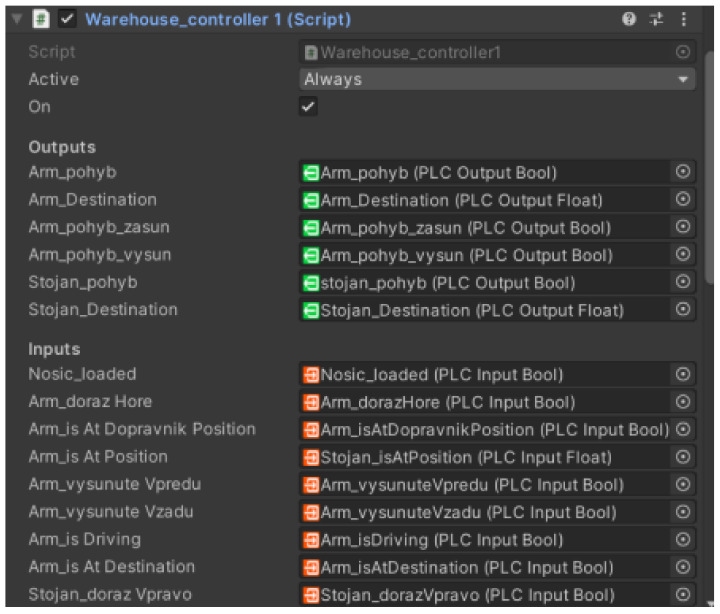
Controlling inputs and outputs.

Game4Automation framework offers scripts to add functionality if the external device is not connected to the simulation, either as an extension of the simulation or for demonstration purposes. We will create a “WarehouseController” (Figure 29) script which will replace the rest of the functionality and thus the simulation of automatic stacking. The script will use the signals from the Unity model but also from the real warehouse system. We will create an automation algorithm, so that the simulation works without user intervention. In this section, we will use the information we have achieved from the previous section and develop an algorithm that will simulate a warehouse stacker in a virtual environment.

#### 5.3.1. Script

First of all, when creating a script, we need to initialise all signals and auxiliary variables. Specifically, we map the input and output signals as objects in the script (Figure 29).

#### 5.3.2. Position Coordinates

**Figure 30:**
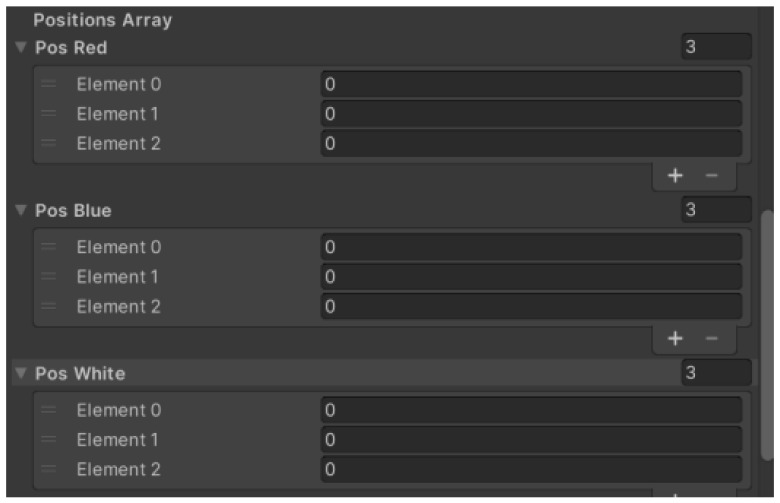
Arrays with positions in the warehouse.

Figure 30 shows the fields of the position in the warehouse. Each row in the warehouse, and therefore a specific workpiece colour, has an associated field. A field of size 3 checks for free or occupied positions and when a workpiece is stacked in a vacant position, the value in the specific field changes to 1 (indicating an occupied position). Empty stock has a value in all positions 0. Table 1 shows the specific coordinates of each position in the warehouse and the positions of the stacker.

#### 5.3.3. Assignment of Workpiece Colour

**Figure 31:**
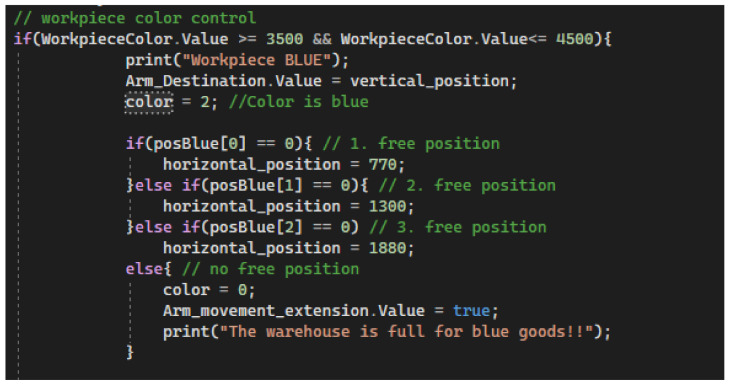
Assignment of workpiece colour.

The first process that is executed when the programme is started is the mode check. The mode is is set to auto-stack. The mode can later be extended to include unloading or manual control of the system. During stacking, it is necessary to determine the colour of the workpiece. From the PLC, the Auxiliary variable is sent to the Unity signal. The value of the signal is compared in the condition to check the colour. If the value is between 3500 and 4500—the workpiece is blue (Figure 31). Red colour is between 1500 and 3500. The white colour of the workpiece is between 50 and 1500. If the sensor senses a value different from the selected colours, the sensed carrier with the workpiece is marked as unknown colour. When a workpiece colour is detected, a free workpiece-stacking position for the specific colour is searched for and the horizontal position of the first free position in the warehouse is stored. This condition is executed first in our algorithm and determines the stacking position for the stacker arm.

#### 5.3.4. Moving the Stacker to the Warehouse

**Figure 32:**
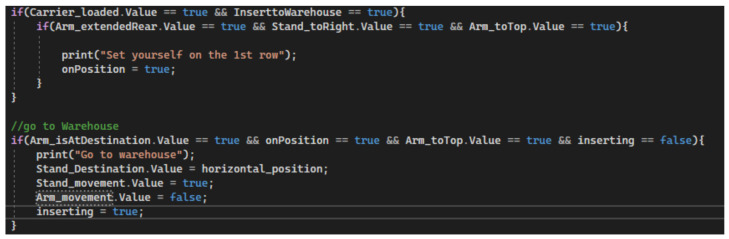
Moving the stacker to the warehouse.

The stacker starts stacking the carrier from the starting position. After the arm has lifted the carrier from the conveyor belt, the arm retracts and sets the carrier back to the initial vertical zero. The algorithm always checks the input signals, in the first condition if the model is in the stacking mode and contains a loaded carrier, the vertical position is assigned to the stacker arm. The second condition also checks if the arm is in the correct position. If the condition is met, the rack will begin to move horizontally into the stacker. The position where the stacker stops is set by the forward coordinate. After checking these two conditions, the arm together with the stacker is at the correct position, ready to load the carrier with the workpiece into the empty position in the warehouse (Figure 32).

#### 5.3.5. Loading the Carrier into the Warehouse

**Figure 33:**
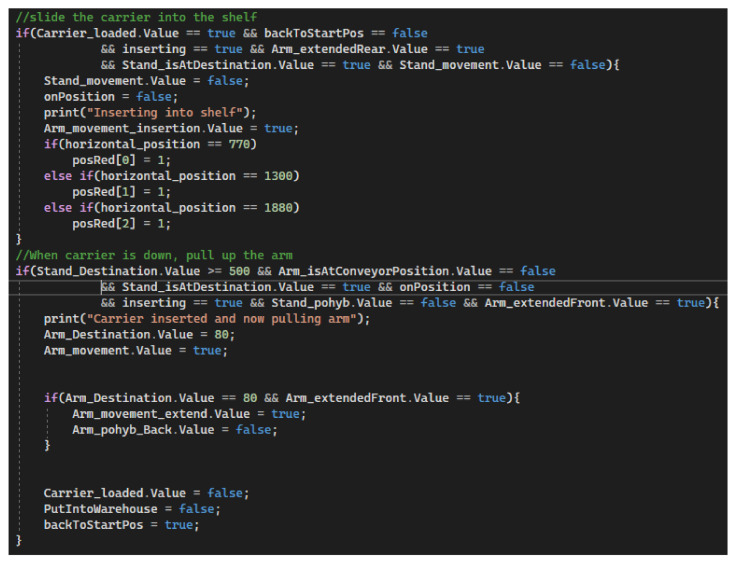
Loading the carrier.

We divide the process of stacking the carrier in a free position in the warehouse into two parts. The first step is to retract the carrier arm. The arm is retracted and stops only at the pressed switch, which indicates that the arm is maximally retracted (in this case in the warehouse). The moment the arm is retracted, the occupied position is also recorded, in a specific field position according to the colour of the workpiece. The second step in the process is to pull the stacker arm out correctly. According to the table with the vertical coordinates, we know to what specific value the arm must be lowered and then extended to the base position. The second condition of the process checks both the input and output signals. If the model is in the correct state, a vertical movement of the arm is performed so that the carrier is put in position and then the arm is returned to the initial position. At the end of the process, the particular auxiliary variables are set to FALSE and a new process is started. This new process—return to the starting position, is triggered by the variable ‘backToStartPos’, which is set set to TRUE Figure 33).

#### 5.3.6. Returning the Stacker

**Figure 34:**
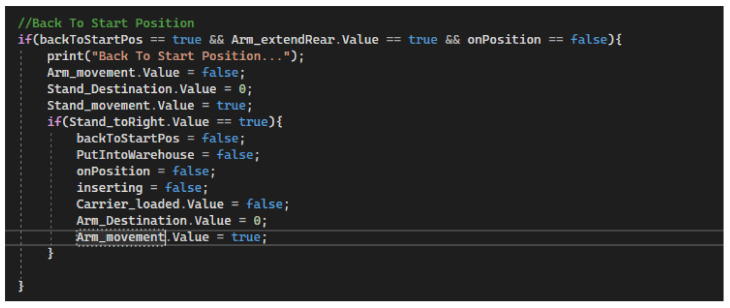
Returning the stacker.

The condition for returning the stacker to the initial position must be met. This process is triggered by the auxiliary variable “backToStartPos”, which checks the state of the stacker. The arm is not extended to the front and the stacker is not yet at the starting position. When the process starts, the horizontal position is set to 0 and the rack and arm is set in motion. When the rack arrives at the selected position, the auxiliary variables are reset and the entire stacking process can be restarted. At this point, a new workpiece carrier can be added to the conveyor belt and the stacking system will work automatically, according to the warehouse status acquired so far (Figure 34).

#### 5.3.7. Input Signals

The conveyor contains a conveyor belt that can move forward and backward. For this function we use the “Drive_Simple” component. The movement of the stacker will be used by the “Drive_Destination Motor” script. Controlled movement of the stacker is different from normal movement in that it is necessary to know the final and current position of the object. In this case, we will use this script, which exactly meets our requirements. We can keep track of when the stacker is in motion, what position it is in, and where it is pointing. The final position is predetermined, the rack will always have specified where it should stop in the warehouse. This position is determined by the available space in the warehouse. We can also check if the stacker is already at the target position. We will use the same script for the vertical movement of the stacker arm. When filing the rack on a shelf in the warehouse, we need to identify a specific position to set the arm to. As for the stacker, the movement of the arm and its target position will be changed and controlled according to the colour of the workpiece.

### 5.4. Running the Simulation

**Figure 35:**
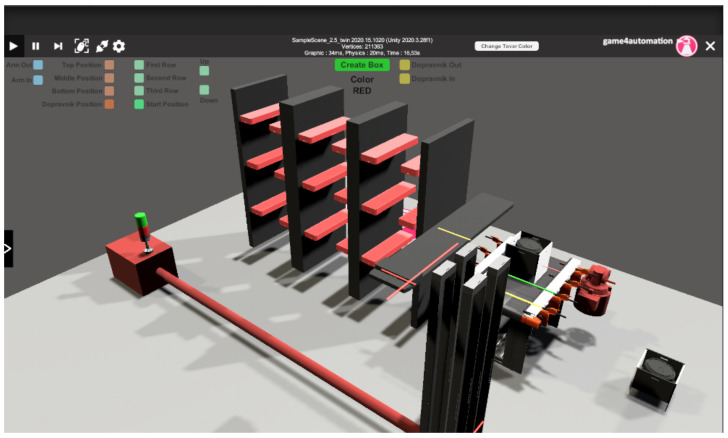
Simulation of the warehouse stacker model.

**Figure 36:**
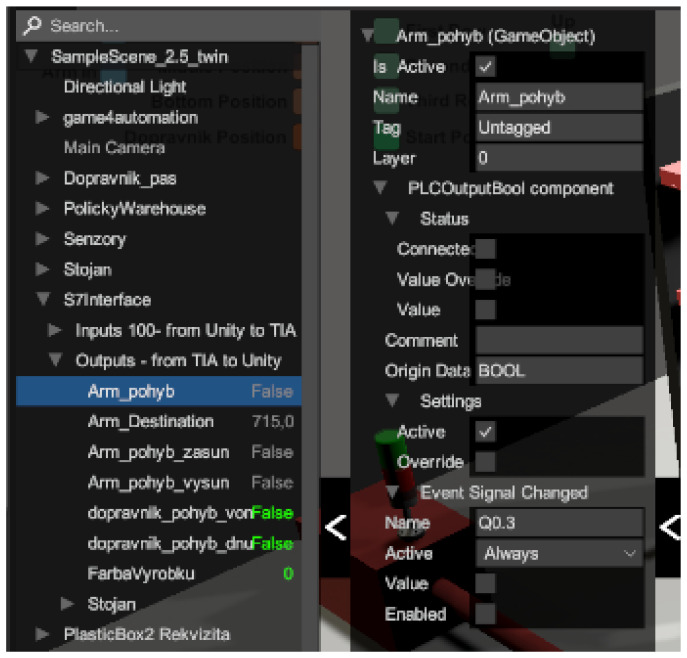
Control panel.

We created a build application in the Unity editor environment (Figure 35). In the main window of the simulation we can connect to the connected PLC device via the Connection icon. If the connection is enabled and functional, the conveyor and the conveyor simulation model will react to signals bilaterally. We can control the real conveyor belt using the signals in the simulation and vice versa. However, we can control the simulation without being connected to a PLC device. We have buttons available for manual control of the conveyor belt, stacker arm, and rack. The button to create a new carrier with a workpiece is used when simulating the stacking of multiple carriers in the warehouse. When the simulation begins, we make sure that the workpiece carrier is ready on the conveyor belt. If the carrier is there, we can start the stacking simulation using the move conveyor belt forward button. When the carrier with the workpiece arrives at the second signal on the conveyor belt, the stacker arm starts to perform the automatic simulation. The user can follow the simulation and can edit objects and signals directly during the simulation. On the left side of the screen, we click the arrow that contains the hierarchy of objects used in the scene. There is also an S7 interface with the signals used. These signals we can set or toggle their state, or we can deactivate them. The add-on improves troubleshooting and makes it easier to simulate different scenarios. Figure 36 shows the output signals and their states directly in the running simulation.

## 6. Conclusions

The aim of this paper is to present a general methodology/method for creating simple and more complex digital twins. A further aim of the paper is to point out the fact the relevant literature presents the concept of digital twin not always in the same way and is misused rather than just a buzzword. In our search, we found that the vast majority of “digital twins” in the analyzed articles did not even meet the first necessary condition—bidirectional data flow between the real system and its digital version. The company IOT Analytics in its study [60] found that companies classify the digital twin in up to 210 different combinations. Because of these facts, we decided to create simple and straightforward-to-understand educational case studies that use available tools and techniques to create basic digital twins that satisfy an essential condition—bidirectional communication between the real system and its digital version. These case studies will form the basis for the creation of pilot courses for Industry 4.0 education and can be further modified for the development of Industry 4.0 educational materials and technical practice. The aim of the first case study was to link the physical model of the assembly line 822 with its digital version and make them interact with each other. We ensured two-way communication between the physical and digital lines, where an event executed on one line was manifested on the other. There are described the affordable tools we used for the development of our application, choosing them in a way that made our work efficient and clear, especially in software.

In our second case study, we focused on a more complex discrete manufacturing system and tested whether it was possible to model a digital twin for such a system. The digital twin was also used for virtual commissioning. During the development of the digital twin in this case study, part of the real storage model was damaged. The digital twin was subsequently used as a virtual extension of the functional part of the real line. This showed the potential of a new application level for digital twins in virtual expanding of real production lines.

The application contributions of presented article can be summarized in the following points:

- **Educational and awareness-raising value of the article consisting in the creation of “real” digital twin**
  As the survey has shown, the vast majority of studies, despite their proclamations, do not really deal with digital twins, but only with models or shadows. We felt it necessary to point this out and to show illustrative examples of real-life digital twins in clear and replicable cases.
- **Low-cost solution for presenting of creation of digital twin**
  Our solution offers cost-effectiveness, which is a crucial aspect compared to other solutions explored in the project. We rely on freely or commonly available technologies.
- **Flexibility ensured by the use of Unity engine**
  It is worth highlighting that the flexibility of the solution presented in this article is due to the use of the 3D engine Unity. Unity offers a variety of tools that enabled the authors to easily create and manipulate 3D models, as well as simulate real-world interactions. The created digital twins may not only be presented on a computer screen, but also on other platforms, such as mixed reality in Microsoft HoloLens 2.
- **Creation of new case studies for pilot projects for the creation of educational materials for the training of engineers for Industry 4.0—pilot Engineer 4.0 programmes**
  The case studies prepared in this article, which integrate operational and information technologies, are well-suited for use in educating engineers on the digitalization of production processes. This effort will complement our previous study [61] and complement the upcoming curriculum for the Industry 4.0 pilot training programs.

In our future plans, we aim to focus on utilizing real CAD models in digital twins. Subsequently, we want to develop a methodology to verify the correct assembly of digital twins by comparing the timestamps of events on the real and virtual lines.

## Figures and Tables

**Table 1:** Table of positions and coordinates.

Position	Coordinate
The X position of the starting position	0
Poloha Y ramena výberu nosiča	715
Y position of carrier selection arm “above”	615
1st warehouse column	710
1st line of “selection”	100
1st line of “deposition”	0
2nd warehouse column	1300
2nd line of “selection”	450
2nd line of “deposition”	350
3rd warehouse column	1880
3rd line of “selection”	830
3rd line of “deposition”	730

## Data Availability

Not applicable.
